# Past and Future Hurricane Intensity Change along the U.S. East Coast

**DOI:** 10.1038/s41598-019-44252-w

**Published:** 2019-05-24

**Authors:** Mingfang Ting, James P. Kossin, Suzana J. Camargo, Cuihua Li

**Affiliations:** 1 Lamont-Doherty Earth Observatory, Columbia University, Palisades, NY 10964 USA; 2 NOAA National Centers for Environmental Information, Center for Weather and Climate, 1225 W. Dayton Street, Madison, Wisconsin 53706 USA

**Keywords:** Atmospheric science, Natural hazards

## Abstract

The ocean and atmosphere in the North Atlantic are coupled through a feedback mechanism that excites a dipole pattern in vertical wind shear (VWS), a metric that strongly controls Atlantic hurricanes. In particular, when tropical VWS is under the weakening phase and thus favorable for increased hurricane activity in the Main Development Region (MDR), a protective barrier of high VWS inhibits hurricane intensification along the U.S. East Coast. Here we show that this pattern is driven mostly by natural decadal variability, but that greenhouse gas (GHG) forcing erodes the pattern and degrades the natural barrier along the U.S. coast. Twenty-first century climate model projections show that the increased VWS along the U.S. East Coast during decadal periods of enhanced hurricane activity is substantially reduced by GHG forcing, which allows hurricanes approaching the U.S. coast to intensify more rapidly. The erosion of this natural intensification barrier is especially large following the Representative Concentration Pathway 8.5 (rcp8.5) emission scenario.

## Introduction

Hurricanes are one of the most devastating and costly natural disasters to befall the United States coastal regions^1^. They account for about 20% of the total billion dollars weather and climate related disasters and over 50% of the total loss in the U.S.^1^. Previous studies^2,3^ have shown that there has been increased destruction associated with Atlantic hurricanes in the past few decades. How human activity may contribute to hurricane intensity change in the future, particularly landfalling hurricanes, is thus an extremely urgent question for society at large. The longer-term change in hurricane intensity in the observational record, however, is somewhat uncertain^4^. The uncertainty is partly due to the lack of long reliable historical hurricane data^5,6^, and partly due to the large natural multidecadal variability in the North Atlantic sea surface temperatures (SST) that are known to affect hurricane activity in the tropical Atlantic^7,8^. Large scale environmental variables, such as SST and vertical wind shear (VWS), are often used as a proxy for hurricane potential intensity (PI)^9,10^ and genesis^11–13^, respectively. The VWS, defined as the magnitude of the vector wind difference between the upper (200 hPa) and lower (850 hPa) troposphere, is well known to affect hurricane activity on various time-scales, with weak VWS favorable for hurricane convective organization and intensification^14–16^. A recent study^17^ noted that hurricane intensification rates near the U.S. southeast coast were suppressed in recent decades due to enhanced VWS. That study, as well as previous studies^18,19^, further noted that there is a large decadal variation in the dominant mode of VWS, which is characterized by a dipole spatial pattern with one node along the U.S. East Coast and one in the tropical North Atlantic, or the hurricane main development region (MDR)^17^. The dipole structure also exists in the SST pattern, with slightly weaker warming in the region of increased VWS and stronger warming in the MDR. During the phase when the tropical Atlantic experiences weak VWS and warm SST, and thus greater hurricane activity in the MDR region, the U.S. coast is shielded from strong landfalling hurricanes due to the strong VWS and weak SST warming along the coastal regions. Thus the dipole mode provides a protective barrier to the U.S. East Coast when hurricanes are particularly active and strong in the tropical Atlantic MDR, which seems to be the case in recent decades. It is not clear, however, whether the dipole pattern and the related recent increases in VWS along the U.S. East Coast are driven entirely by natural decadal variability, or there may be contributions from anthropogenic forcing.

Furthermore, it is essential to understand how landfalling hurricanes along the U.S. East Coast may change in the future as greenhouse gas (GHG) forcing increases. While it is certain that SST will continue to rise as a result of GHG forcing, it is not clear how VWS may respond to GHG forcing along the coastal regions. Using the IPCC AR4 21^st^ Century projection of the tropical Atlantic VWS changes, Vecchi and Soden^20^ projected an increase in VWS in the Atlantic MDR region by the end of the 21^st^ Century. They further attributed the VWS increase to the weakened Pacific Walker Circulation. Similar changes have been noted for the Coupled Model Intercomparison Project Phase 5 (CMIP5) models^21^. In this study, we focus on the future changes in VWS in terms of their projection onto the VWS dipole pattern between the U.S. East Coast and the tropical Atlantic MDR^17^ in the current climate. The main goal here is to distinguish the contribution to the dipole VWS pattern from anthropogenic forcing and natural decadal and multidecadal variability and the implication to hurricane intensification along the U.S. East Coast in the future.

We examine the change in both hurricane potential intensity (PI) and VWS due to anthropogenic changes using CMIP5 coupled ocean atmosphere simulations as well as the Community Earth System Model Large Ensemble (CESM-LE)^22^, in the presence of the background natural multidecadal variability. We address two related questions here: is the change over the past 70 years in hurricane PI and VWS along the U.S. East Coast dominated by anthropogenic forcing or natural multidecadal variability, and how would the future changes of these conditions impact hurricane intensity and formation in the region?

## Results

### Causes of the dipole mode

**Figure 1:**
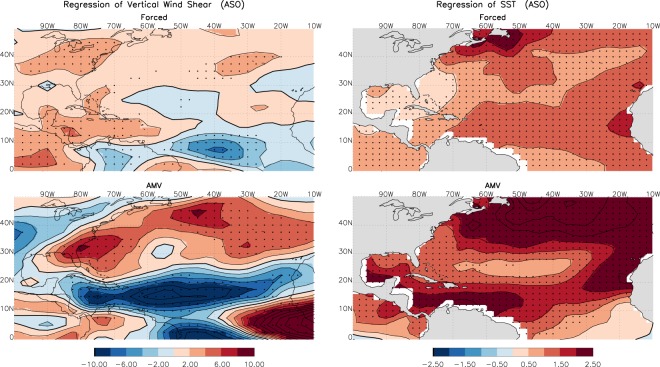
Hurricane peak season (August, September, and October, ASO) vertical wind shear (VWS, left) and sea surface temperature (SST, right) regressed onto the radiatively forced (top) and AMV (bottom) indices as shown in Supplementary Fig. S1, with the forced component obtained through the Signal-to-Noise Maximizing EOF analysis PC1 from CMIP5 multi-models (see methods section). The winds and SST are taken from the NCEP/NCAR reanalysis and NOAA ERSST, respectively, for the period 1948 to 2015. Units are m/s per °C of SST index for VWS and °C per °C of SST index for SST. The stippling indicates statistical significance of the regression coefficients at the 5% level using 2-sided Student’s t-test.

We first isolate the VWS and SST patterns that are externally forced due to radiative forcing change from those due to natural climate variability. Figure 1 shows the observed VWS and SST linearly regressed to the external radiative forcing component and the Atlantic Multidecadal Variability (AMV) for the period 1950 to 2015. The external radiative forcing and AMV here are defined using the signal-to-noise maximizing empirical orthogonal function (SNEOF) analysis^23^ performed on the global SST from the CMIP5 multi-model ensembles^24,25^. The SNEOF for separating the forced and AMV time series are described in the methods section and the corresponding time series are shown in Supplementary Fig. S1. We also confirm the results in Fig. 1 using the CESM-LE historical simulations with a total of 42 ensemble members started from slightly different initial conditions^22^ (see Supplementary Fig. S2). Results in Fig. 1 (and that in Supplementary Fig. S2) indicate that the observed VWS dipole pattern during the peak hurricane season (August, September and October, or ASO), with enhanced shear along the U.S. East Coast and suppressed shear in the tropical North Atlantic, is primarily due to the positive phase of AMV. The contribution from anthropogenic and natural radiative forcing to the dipole pattern is rather weak during this period. The SST anomalies in Fig. 1 indicate a basin-wide warming due to anthropogenic forcing and a horseshoe pattern with stronger warming in the tropical and midlatitude North Atlantic compared to that in the subtropical North Atlantic during AMV positive phase. Previous studies have shown that the warmer the SST the stronger the hurricane PI^8–10^. Thus Fig. 1 implies that when anthropogenic warming is combined with the positive phase of AMV, PI increases in the entire tropical North Atlantic. While the suppressed VWS in MDR enhances the possibility of hurricane formation and intensification, the stronger VWS along the U.S. East Coast suppresses the intensification rate in that region, consistent with Kossin^17^. In contrast, when the anthropogenic warming is combined with the negative phase of AMV, the SST warming in the basin is slightly dampened while the intensification rate along the U.S. coast is enhanced due to the suppressed VWS. In summary, AMV modulates considerably the hurricane activity in both the tropical North Atlantic where hurricanes often form, and the U.S. East Coast where hurricanes make landfall. When hurricane activity is enhanced in the MDR region during the positive phase of AMV, the intensification along the U.S. East Coast is often suppressed due to stronger VWS and less SST warming there, and vice versa. Thus the AMV-related dipole pattern in VWS and SST provides a natural protective barrier for the U.S. East Coast from strong hurricane intensification and landfalls.

### VWS forced by future anthropogenic radiative forcing

**Figure 2:**
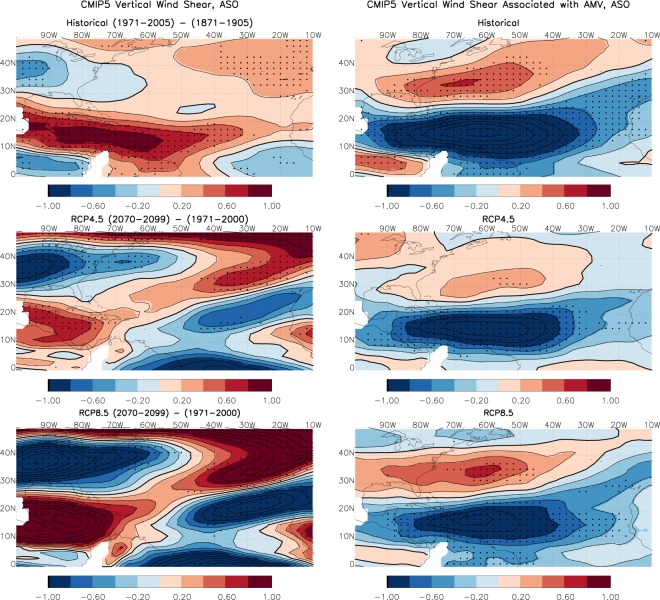
Vertical wind shear from left panels: CMIP5 multi-model mean representing the radiatively forced changes between two different time periods for historical (top, 1971–2005 minus 1871–1905), rcp4.5 (middle, 2070–2099 minus 1971–2000) and rcp8.5 (bottom, 2070–2099 minus 1971–2000) and right panels: composite based on positive AMV years minus negative AMV years for the historical, rcp4.5 and rcp8.5 integrations. Units are in m/s and the stippling indicates 75% of the models or ensemble members having the same sign changes.

The VWS has not been substantially influenced by the anthropogenic radiative forcing in the past few decades (see top left panel in Fig. 1). However, as GHG forcing increases, the VWS associated with anthropogenic forcing may also increase in amplitude. To explore future changes in VWS and its relative role as compared to the AMV, Fig. 2 shows the CMIP5 multi-model mean (MMM) changes in VWS as the difference between two time periods, between 1971–2005 and 1871–1905 for the historical period, and between 2070–2099 and 1971–2000 for the two future scenarios (rcp4.5 and rcp8.5). As a comparison, we also average the periods identified as positive and negative AMV (defined as one standard deviation above or below the AMV index), and take the difference between the positive and negative phases to illustrate the VWS pattern associated with AMV. It is clear that the VWS patterns simulated by the model associated with AMV (right panels of Fig. 2) have a similar dipole structure to observations (lower left panel in Fig. 1), with enhanced VWS along the U.S. coast and suppressed VWS in the tropical Atlantic MDR for positive AMV. The radiatively forced VWS change, however, paints a very different picture. Along the U.S. East Coast, the VWS shows an opposite sign to that corresponding to positive AMV, with suppressed VWS along the coastal region. In the tropical North Atlantic extending from the Caribbean to the Gulf of Mexico, the VWS is enhanced. However, the enhanced VWS in the tropical Atlantic is only limited to the western part of the MDR, with the exception of the historical period, when the intensified VWS extends to the central tropical Atlantic including most of the MDR region. The differences between historical and future scenarios may be linked to aerosol forcing, which is relatively strong during the historical period^8^. The forced patterns with enhanced VWS in the Caribbean region are somewhat similar to those shown in CMIP3 models^20^ and a subset of the CMIP5 models^21^. But the strong suppressed shear along the U.S. East Coast, although present in the CMIP3 models, is much stronger in Fig. 2, particularly for the rcp8.5 future scenario. The implication of the anthropogenically forced future VWS suppression along the U.S. East Coast is that future hurricanes may go through stronger intensification and cause more powerful destructions when moving into the coastal region.

### Relative amplitude of VWS due to climate change and AMV

**Figure 3:**
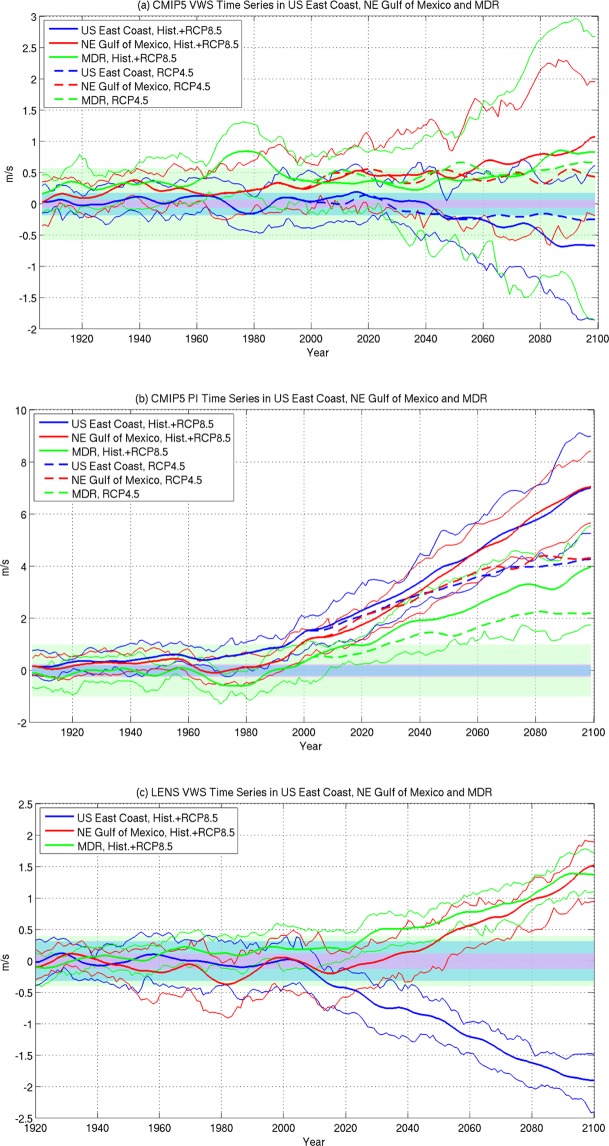
(**a**) CMIP5 multi-model mean vertical wind shear for historical + rcp8.5 (thick solid lines), historical + rcp4.5 (thick dashed lines), and the 25% and 75% of the model spread using rcp8.5 (light solid lines) averaged over the U.S. East Coast (25N–40N, 80W–60W, blue), Gulf Coast (20N–30N, 95W–82W, red), and the Main Development Region (10N–20N, 80W–20W, green). (**b**) same as in (**a**), but for CMIP5 MMM tropical cyclone potential intensity averaged over the same three regions. (**c**) CESM-LE 42 ensemble member mean VWS (solid lines) and the 25% and 75% ensemble spread (light solid lines) averaged over the same three regions. Shadings in all three panels indicate the amplitude of the VWS or potential intensity associated with average AMV in the U.S. East Coast (light blue), Gulf Coast (purple) and MDR (light green).

In the future, AMV modulation of the tropical cyclone activity as seen during the historical period should continue and the relative amplitude of the anthropogenically forced change and the AMV could help determine the projections of future hurricane activity. This relative relationship is illustrated in Fig. 3a which shows the area averaged VWS for three geographical regions, the U.S. East Coast (25N–40N, 80W–60W), the Gulf Coast (20–30N, 95W–82W), and the MDR, for 1900 to 2100. The future anthropogenic emissions following both the rcp4.5 and rcp8.5 are included. The VWS associated with AMV is also indicated in Fig. 3a which illustrates the amplitude range of VWS due to AMV. While the magnitude of the MMM VWS associated with anthropogenic forcing is largely within the natural variability for the historical period, the decrease in the U.S. East Coast VWS (increase in the Gulf Coast) starts to dominate over the AMV VWS in the second half of the 21^st^ century. By the end of the 21^st^ century, the forced VWS in the coastal regions is almost twice as strong as that associated with the positive or negative phases of AMV, making possible for hurricanes to intensify faster along the U.S. East Coast (slower along the Gulf Coast). In the MDR, the VWS associated with AMV is rather large, and the increase in forced VWS only starts to dominate at the end of the 21^st^ Century. The model spread of the VWS is fairly large, indicated by the light solid lines showing the 25th to 75th percentile of models’ values. But for the U.S. East Coast, the majority of the models indicate decreased VWS with magnitude ranging from +0.5 m/s to −2 m/s at the end of the 21^st^ Century. Figure 3c shows the VWS change in the CESM-LE. The ensemble spread in the single model is clearly much smaller than the multi-model spread in Fig. 3a, with magnitude ranging from −1.5 m/s to −2.5 m/s at the end of the 21^st^ Century. The VWS change in the CESM-LE is at the high end of the model spectrum as can be seen comparing Fig. 3a,c. The MDR VWS changes show the largest multi-model spread and a relatively small amplitude. The large spread in this case is not surprising, as the spatial pattern in Fig. 2 indicates a nodal point of VWS change in the MDR. The Gulf Coast shows an increase in VWS with the multi-model magnitude ranging from −0.5 m/s to +2 m/s. In the CESM-LE, both the MDR and the Gulf Coast show a similar magnitude increase in VWS with an ensemble range from +1 m/s to +2 m/s.

Due to the general increase in SST in the future scenario simulations, the hurricane PI increases across the North Atlantic domain^8^. Figure 3b shows the CMIP5 MMM PI time series averaged for the same three geographic regions. There is a monotonic increase in PI for all three regions, but the increase is much faster for both the U.S. East Coast and the Gulf Coast compared to the MDR, consistent with Ting *et al*.^8^. For the PI, the multi-model spread is not as large as for VWS, showing a more robust SST warming in all models – the main driver of the PI change. The fast increase in PI along the U.S. East Coast, coupled with the more favorable wind shear environment for hurricane intensification there, suggests that hurricanes tracking toward the U.S. East Coast will have a better chance of achieving its PI in the future, which may be much stronger than what we’ve experienced in the past. For the Gulf Coast, the two effects, warmer SST and stronger wind shear, oppose each other. How future hurricane intensity may change there will depend on the relative influence of the two factors, leaving future projections of hurricane intensity along the Gulf Coast less certain.

### Time of emergence of forced VWS signal relative to AMV

The time when anthropogenically forced VWS may be strong enough to erode the natural AMV-induced dipole VWS pattern in the future is of particular interest, as the AMV dipole pattern provides a natural protection to the U.S. East Coast hurricane intensification during active MDR tropical cyclone epochs. Based on CMIP5 MMM, the anthropogenic forced VWS will reach a similar amplitude as that due to AMV by around 2050. However, the multi-model spread is large and thus the time of emergence differs widely from model to model. Using Fig. 3c, the CESM-LE VWS change indicates that by 2020, the anthropogenic forced VWS will reach the same amplitude as that due to AMV, and by 2050, the forced VWS will be twice as strong as that associated with AMV. This suggests that anthropogenic forcing will substantially erode the natural protection of AMV to the U.S. East Coast hurricane activity by 2050. The AMV amplitude is relatively weak in the Gulf Coast region, thus anthropogenic forcing will not substantially change the natural variability of the hurricanes moving into that region. There, as shown earlier, the relative role of the increasing PI and enhanced VWS may cancel out some of the future changes in hurricane intensity and which factor may prevail is an interesting question to explore further. For the MDR region, where AMV amplitude is rather large, and the anthropogenic influence on PI and VWS also oppose each other, we expect that the natural variability of hurricane activity may continue to dominate into the future.

### Causes of future VWS change

**Figure 4:**
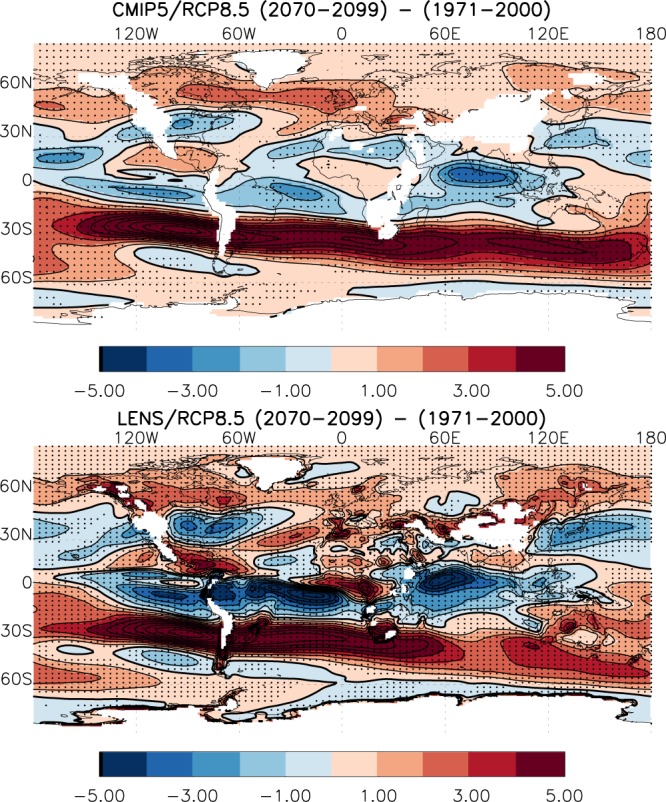
Global pattern of VWS difference between 2070–2099 and 1971–2000 for CMIP5 MMM (top) and CESM-LE 42 ensemble mean (bottom) under the rcp8.5 scenario. Units are in m/s and the stippling indicates 75% of the models or ensemble members having the same sign changes.

The change in tropical wind shear under GHG forcing was attributed to changes in the tropical Walker circulation, the east-west overturning circulation, in Vecchi and Soden^20^. Figure 4 shows the VWS change between end of the 21^st^ Century and the end of the 20^th^ Century under rcp8.5 scenario for both CMIP5 MMM and CESM-LE. The reduced wind shear along the US East Coast under future GHG forcing is consistent with the expansion of the Hadley circulation^26,27^, and the related northward shift of the midlatitude jet stream in both the CMIP5 MMM and the CESM-LE. To determine the change in VWS due to contributions from the upper and lower levels, Supplementary Fig. S3 shows the wind vector differences between the two periods along with the climatological wind magnitude at 200 hPa and 850 hPa for CMIP5 MMM and CESM-LE. The northward shift of the jet is clearly seen at both the upper and lower levels and in the Atlantic and the Pacific regions. There is also a clear indication of a southward flow at the lower level and northward flow at the upper level, implying an enhanced and northward extended Hadley circulation, at least in the tropical North Atlantic. Supplementary Fig. S3 strongly suggests that not only the VWS decreases along the U.S. East Coast, but this is also the case for the East Coast of Asia, where one might expect that in the future typhoons would intensify more than in the historical climate and reach higher potential intensity values.

## Summary

Our results emphasize the potential threat that hurricanes may become more intense in the future as they move toward the East Coast of the United States. This is mainly caused by a reduction of future vertical wind shear along the U.S. East Coast, which favors the intensification of hurricanes by less dispersion of latent heat energy, compared to the case of strong VWS. The weak vertical wind shear environment would help hurricanes reach their potential intensity, the maximum possible intensity given the environmental conditions, which has already increased in magnitude due to warmer sea surface temperatures. Furthermore, the modulation due to natural multidecadal climate variability in the North Atlantic, the AMV, may become less effective in the future as GHG forced changes become more dominant. In the recent past, AMV modulation dominated the VWS pattern, which favors a dipole structure with one node in the MDR region and another along the US East Coast with opposite sign anomalies. When VWS is weak in the MDR region, which favors more hurricane activity in the tropical North Atlantic, the VWS along the US East Coast is suppressed. This dipole pattern provides a natural barrier to strong hurricane occurrence and intensification along the East Coast. As shown in Kossin^17^, the decades since the early 90s have seen suppressed hurricane intensification along the U.S. East Coast even when hurricane activity is heightened in the tropical Atlantic. Our results indicate that natural multidecadal variability, through the dipole mode modulating the vertical wind shear and the hurricane intensification rate, is largely responsible for the protective barrier of the recent East Coast hurricane activity. In the future, the role of the AMV modulation may diminish in comparison to the ever stronger anthropogenic GHG forcing. Thus the protective barrier of the AMV to the East Coast hurricane intensification may be largely eroded by the GHG effect. Coupled with the robust warming of the ocean surface temperature in the future, it is likely that the US East Coast will experience unprecedented hurricane intensification in the future, causing even greater threats to the coastal community. For the Gulf Coast, the future projected increase in VWS may make the region slightly less vulnerable to strong landfalling hurricanes, although the increase in potential intensity there may outweigh the VWS effect.

## Methods

### Observed and modeled SST and VWS

The observed sea surface temperature used in this study is taken from the National Oceanic and Atmospheric Administration (NOAA) Extended Reconstructed SST version 3b (ERSST v3b) monthly data^28^ and the model SST data are directly taken from the CMIP5 and CESM-LE monthly outputs. The vertical wind shear used in this study is computed from the difference in vector wind at 200 hPa minus that at 850 hPa for both observations and climate model outputs. For observations, VWS is computed as in Kossin^17^, that uses the daily-mean vector winds from the National Center for Environmental Prediction/National Center for Atmospheric Research (NCEP/NCAR) reanalysis data^29^ from 1948 to 2015. The daily vertical wind shear was computed first and then averaged to get the monthly and seasonal mean VWS used in the analysis. For the CMIP5 models and the CESM-LE, VWS was computed from the monthly mean vector winds from the model output for each model ensemble member. The resulting monthly mean VWS are found to be relatively insensitive to whether one uses daily or monthly vector winds for the vertical shear calculation. The multi-model or multi-ensemble mean and spread are then obtained from the 27 CMIP5 simulations (see Supplementary Table S1) and the 42-member CESM-LE, respectively.

### Hurricane potential intensity

The hurricane potential intensity used in this study is as computed in Ting *et al*.^7^ and using the definition developed by Bister and Emanuel in a series of papers^8,30–33^. The PI is defined as

1 $$PI=\frac{{C}_{k}}{{C}_{d}}\frac{{T}_{s}}{{T}_{0}}(CAP{E}^{\ast }-CAP{E}^{b})$$

where C_k_ is the surface exchange coefficient for enthalpy and C_d_ the drag coefficient, T_s_ is the SST and T_0_ the air temperature at the outflow level. The convective available potential energy (CAPE) is the vertical integral of parcel buoyancy as a function of temperature, pressure, and specific humidity, as well as the vertical profile of virtual temperature. CAPE* is the value of CAPE for an air parcel lifted from a saturated state at the sea surface temperature while CAPE^b^ is the value of CAPE calculated from the actual humidity value, both at the radius of maximum wind. The environmental variables used to calculate PI include the SST, sea level pressure, and vertical profiles of temperature and specific humidity.

### Signal-to-noise maximizing empirical orthogonal function analysis (S/N EOF)

In order to separate the radiatively forced and naturally occurring multidecadal variations in VWS and SST, we apply the S/N maximizing EOF analysis^23–25^ to the global SST from multi-model CMIP5 or CESM-LE outputs. The S/N EOF method first applies a spatial pre-whitening transformation to the model SST aimed at removing the spatially coherent structure due to the internally-generated climate variability, which is defined by the modes of variability contained in the control simulations of the same set of models with pre-industrial radiative forcing. Thus the spatial and temporal variations in the ensemble average SST are purely caused by external radiative forcing after the pre-whitening. An EOF analysis is then applied to the pre-whitened multi-model ensemble average global SST and the first mode usually contains over 80% of the total forced variance. The first principal component (PC1) is then taken as representing the radiatively forced SST changes. The S/N EOF analysis here is applied to the ASO seasonal mean SST to extract the forced signal for the peak hurricane season. The North Atlantic SST in observations or each ensemble member at each grid point can then be separated into two components as follows:

2 $${{\rm{SST}}}_{{\rm{i}},{\rm{j}}}({\rm{t}})={{\rm{a}}}_{{\rm{i}},{\rm{j}}}\,{\rm{PC}}1({\rm{t}})+{{\rm{\varepsilon }}}_{{\rm{i}},{\rm{j}}}({\rm{t}})$$

where SST_i,j_(t) is the observed or modeled ASO seasonal mean SST at time t and grid point (i, j), α_i,j_ is the regression coefficient between SST at that grid point and PC1, and ε_i,j_(t) is the residual SST variation that is not explained by PC1. The North Atlantic domain averaged SST from the first term on the right side of Eq. (2) forms the forced SST index and the second term the internally varying SST index. We apply a 10-year Butterworth filter to the SST data prior to the S/N EOF analysis, thus the internal variability component only contains those with time scale longer than 10-years, and is called Atlantic Multidecadal Variability, or AMV component, as shown in Supplementary Fig. S1 for observed SST. These decompositions can be done for each model and each ensemble member. The spatial patterns of VWS, SST or hurricane PI can be obtained by regressing these fields onto the forced and AMV component of the corresponding observations or model ensembles.

## Supplementary information

Supplementary Figures and Tables

## Data Availability

NCEP/NCAR reanalysis, NOAA ERSST (v3b), CMIP5 models and CESM-LE are all publicly available. The scripts used to calculate PI are publicly available at Kerry Emanuel’s website: ftp://texmex.mit.edu/pub/emanuel/TCMAX/.
