# Supplementary material for: Past and Future Hurricane Intensity Change along the U.S. East Coast
Source: Sci Rep. 2019 May 24;9:7795. doi: 10.1038/s41598-019-44252-w (PMC6534560; doi:10.1038/s41598-019-44252-w)
Supplement: Supplementary file 1 — Supplementary Figures and Tables [file 41598_2019_44252_MOESM1_ESM.pdf]

Supplementary Figures and Tables for

Past and Future Hurricane Intensity Change along the U.S. East Coast

Mingfang Ting[1*], James P. Kossin[2], Suzana J. Camargo[1] and Cuihua Li[1]

**Corresponding author (ting@ldeo.columbia.edu, 845-365-8374)*

[1]Lamont-Doherty Earth Observatory, Columbia University, Palisades, NY 10964, USA

[2] NOAA National Centers for Environmental Information, Center for Weather and Climate, 1225 W. Dayton Street, Madison, Wisconsin 53706, USA

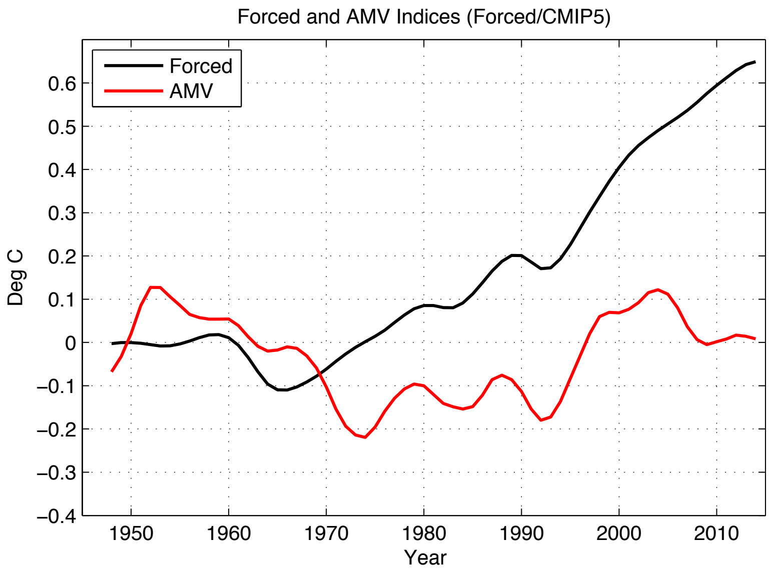

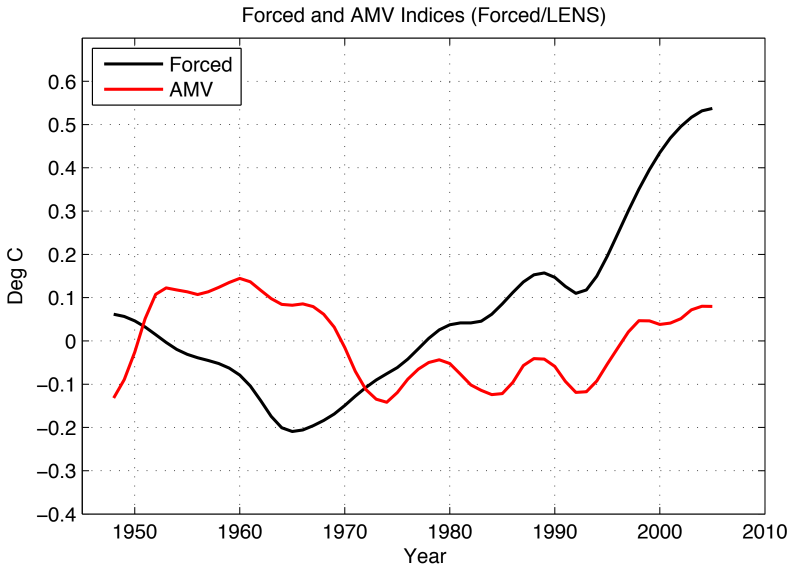

Supplementary Figure S1. Forced and natural AMV time series from observations based on the NOAA ERSST for the August, September, October (ASO) season in the North Atlantic. The time series are obtained using the regression of grid point SST to the Signal-to-Noise maximizing Empirical Orthogonal Function analysis Principal Component 1 (PC1) of the CMIP5 multi-models (top) and the CESM-LE (bottom), and constructed using Eq. 2 in the Methods section.

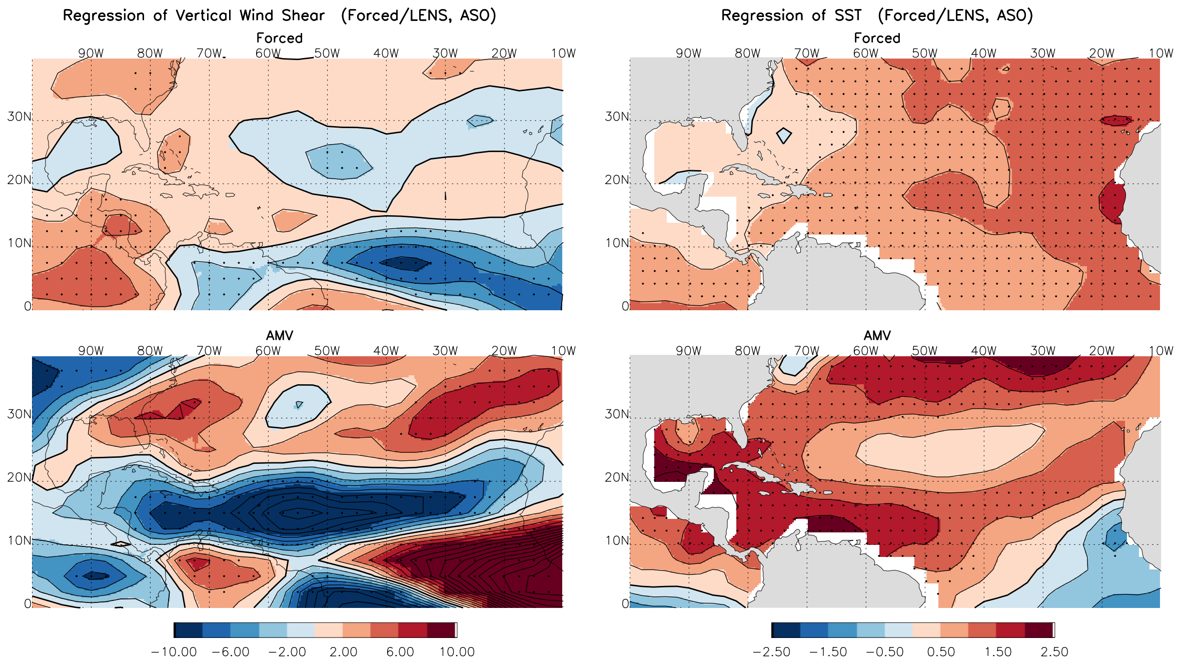

Supplementary Figure S2. Hurricane peak season (ASO) average vertical wind shear (left) and sea surface temperature (right) regressed onto the radiatively forced (top) and natural AMV (bottom) indices as shown in Fig. S1 based on the CESM-LE. Units are m/s per ^{o}C of SST index for VWS and ^{o}C per ^{o}C of SST index for SST. The stippling indicates statistical significance of the regression coefficients at the 5% level using 2-sided Student's t-test.

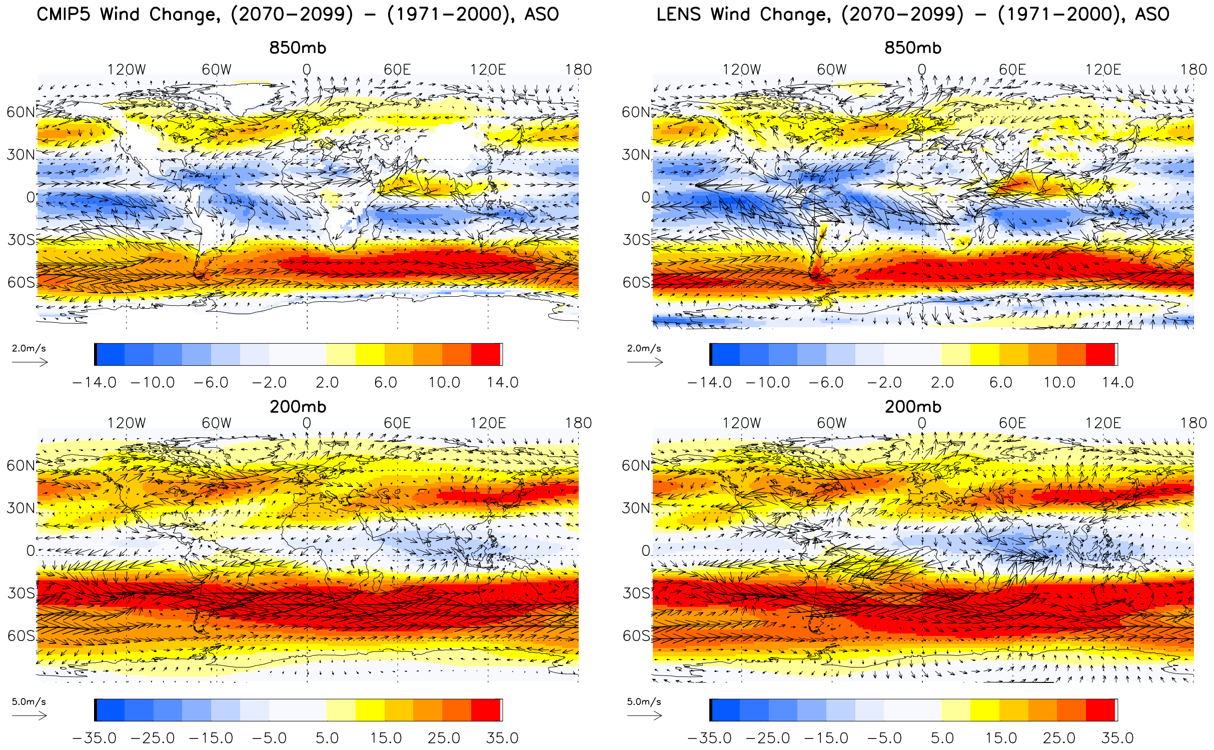

Supplementary Figure S3. The ASO mean 850 hPa (top) and 200 hPa (bottom) horizontal wind vector differences between the average for the future period 2070-2099 following rcp8.5 emissions pathway and the historical period 1971-2000 with historical radiative forcing from CMIP5 multimodel mean (left) and CESM-LE (right). Color shading indicates the climatological zonal wind for the historical period to indicate the climatological zonal jet locations.

Supplementary Table S1. CMIP5 models used in this study with information on host institute, resolutions (L refers to number of vertical levels, T to triangular truncation and C to cubed sphere) and ensemble size.

Institute	Model	Resolution (lon x lat), level	Ensemble size		
			20thC	rcp45	rcp85
Commonwealth Scientific and Industrial Research Organisation (CSIRO),and Bureau of Meteorology, Australia (BOM)	1. ACCESS1-0	N96 (1.25° x 1.875°), L38	1	1	1
	2. ACCESS1-3	N96 (1.25° x 1.875°), L38	1	1	1
Beijing Climate Center (BCC)	3. bcc-csm1-1	T42, L26	3	1	1
Canadian Centre for Climate Modeling and Analysis (CC-Cma)	4. CanESM2	T63 (1.875°x1.875°), L35	5	5	5
National Center for Atmospheric Research (NCAR)	5. CCSM4	288x200 (1.25°x0.9°), L26	6	6	6
Community Earth System Model, Contributors (NSF-DOE-NCAR)	6. CESM1-WACCM	2.5° x 1.9°, L66	1	1	1
Centre National de Recherches Meteorologiques / Centre Europeen de Recherche et Formation Avancees en Calcul Scientifique (CNRM-CERFACS)	7. CNRM-CM5	T127(1.4°x1.4°), L31	10	1	3
Commonwealth Scientific and Industrial Research Organisation in collaboration with the Queensland Climate Change Centre of Excellence (CSIRO-QCCCE)	8. CSIRO-Mk3-6-0	T63(1.875°x1.875°), L18	10	5	5
Institute of Atmospheric Physics, Chinese Academy of Sciences and Tsinghua University (LASG-CESS)	9. FGOALS-g2	128x60, L26	4	1	1
The First Institute of Oceanography, State Oceanic Administration	10. FIO-ESM	T42, L26	3	3	3
Geophysical Fluid Dynamics Laboratory (NOAA GFDL)	11. GFDL-CM3	C48 (2.5°x2.0°), L48	5	1	1
	12. GFDL-ESM2M	144x90 (2.5°x2.0°), L24	1	1	1
NASA Goddard Institute for Space Studies (NASA GISS)	13. GISS-E2-H	2.5°x2°, L40	6	5	2
	14. GISS-E2-R	2.5°x2°, L40	6	5	2
Met Office Hadley Centre	15. HadGEM2-CC	N96, L38	1	1	1
	16. HadGEM2-ES	N96, L38	4	1	4
Institute for Numerical Mathematics (INM)	17. inmcm4	2.0° x 1.5°, L21	1	1	1
Institut Pierre-Simon Laplace (IPSL)	18. IPSL-CM5A-LR	3.75°x1.875°, L39	5	4	1
	19. IPSL-CM5A-MR	2.5°x1.25°, L39	1	1	1
	20. IPSL-CM5B-LR	96x96 (3.75°x1.875°) , L39	1	1	1
Atmosphere and Ocean Research Institute (The University of Tokyo), National Institute for Environmental Studies, and Japan Agency for Marine-Earth Science and Technology (AORI/NIES/JAMSTEC)	21. MIROC5	T85, L40	3	1	1
	22. MIROC-ESM	T42, L80	3	1	1
	23. MIROC-ESM-CHEM	T42, L80	1	1	1
Max Planck Institute for Meteorology (MPI-M)	24. MPI-ESM-LR	T63, L47	3	3	3
	25. MPI-ESM-MR	T63, L95	3	3	1
Meteorological Research Institute (MRI)	26. MRI-CGCM3	TL159 (1.125°x1.125°), L48	3	1	1
Norwegian Climate Centre (NCC)	27. NorESM1-M	144x96 (2.5°x1.875°), L26	3	1	1
